# Effect of Natural Aging on Oak Wood Fire Resistance

**DOI:** 10.3390/polym13132059

**Published:** 2021-06-23

**Authors:** Martin Zachar, Iveta Čabalová, Danica Kačíková, Tereza Jurczyková

**Affiliations:** 1 Department of Fire Protection, Faculty of Wood Sciences and Technology, Technical University in Zvolen, T. G. Masaryka 24, 960 53 Zvolen, Slovakia; zachar@tuzvo.sk (M.Z.); kacikova@tuzvo.sk (D.K.); 2 Department of Chemistry and Chemical Technologies, Faculty of Wood Sciences and Technology, Technical University in Zvolen, T. G. Masaryka 24, 960 53 Zvolen, Slovakia; 3 Department of Wood Processing, Czech University of Life Sciences in Prague, Kamýcká 1176, 16521 Praha 6-Suchdol, Czech Republic; jurczykova@fld.czu.cz

**Keywords:** oak wood, historical wood, chemical composition, flame ignition temperature, spontaneous ignition temperature, activation energy, mass burning rate

## Abstract

The paper deals with the assessment of the age of oak wood (0, 10, 40, 80 and 120 years) on its fire resistance. Chemical composition of wood (extractives, cellulose, holocellulose, lignin) was determined by wet chemistry methods and elementary analysis was performed according to ISO standards. From the fire-technical properties, the flame ignition and the spontaneous ignition temperature (including calculated activation energy) and mass burning rate were evaluated. The lignin content does not change, the content of extractives and cellulose is higher and the content of holocellulose decreases with the higher age of wood. The elementary analysis shows the lowest proportion content of nitrogen, sulfur, phosphor and the highest content of carbon in the oldest wood. Values of flame ignition and spontaneous ignition temperature for individual samples were very similar. The activation energy ranged from 42.4 kJ·mol^−1^ (120-year-old) to 50.7 kJ·mol^−1^ (40-year-old), and the burning rate varied from 0.2992%·s^−1^ (80-year-old) to 0.4965%·s^−1^ (10-year-old). The difference among the values of spontaneous ignition activation energy is clear evidence of higher resistance to initiation of older wood (40- and 80-year-old) in comparison with the younger oak wood (0- and 10-year-old). The oldest sample is the least thermally resistant due to the different chemical composition compared to the younger wood.

## 1. Introduction

When using wood as a structural element, especially as a part of the building structure, it is necessary to assess it from the point of view of fire safety of buildings. It can be realized by using Eurocodes [1]. An important part of ensuring the fire safety of buildings is knowledge of the burning process and forecasting the dynamics of the development of internal fire. Knowledge of fire dynamics is an important starting point, for example, in building design, controlled evacuation, physicochemical and mathematical description of fire–materials and fire–human organism interactions, in the process of determining the causes of fire in finding possible scenarios of fire origin and development, and determining the most similar cause of fire [2].

A significant factor affecting burning rate is chemical composition [3,4,5,6,7,8], primarily the lignin content [3,7,9,10], the species of the wood [3,4,11,12,13], density, moisture content, permeability, anatomy [3] and, last but not least, the aging process [14,15,16]. According to Reinprecht [17], wood decomposition is caused by a complex of chemical reactions associated with mass and heat transfer processes. The wood is ignited when sufficient initial thermal energy is supplied (approximately 104 W·m^−2^), while the specific temperature at which the wood ignites is between 250 and 400 °C.

Activation energy has a great influence on the combustion process. There are several methods to calculate the activation energy. Simple methods (e.g., Arrhenius equation) to much more complex methods (e.g., Ozawa–Flynn–Wall, Kissinger–Akahira–Sunos or Friedman, ASTM E698–18) requiring the results of thermogravimetry (TG), differential thermogravimetry (DTG) and differential scanning calorimetry (DSC), and other progressive instruments and methodologies result in calculating the activation energy and the combustion process kinetics in a complex and precise way. The progressive approaches to the analysis of biomass combustion kinetics are also evident in the study of Majlingová et al. [18]. Based on the flash ignition temperature and spontaneous ignition temperature, according to the ISO 871:2006, the relative comparison of a material resistance against ignition can be carried out.

Several authors studied the thermal properties of wood. They preferred the Arrhenius equation to calculate the activation energy of woody biomass. Martinka et al. [19] focused on the influence of spruce wood form on ignition activation energy and they also investigated the impact of heat flux on fire risk of the selected samples. Rantuch et al. [20] used the Arrhenius equation to calculate the ignition activation energy of materials based on polyamide. In another study, Martinka et al. [21] carried out research on initiatory parameters of poplar wood (*Populus tremula* L.). These initiatory parameters (the critical heat flux density and the surface temperature at the time of initiation) were set on a conical calorimeter using a testing procedure in accordance with ISO 5660-1:2015. Luptáková et al. [22] clarified a comparison of activation energies of thermal degradation of heat-sterilized silver fir wood samples to larval frass in terms of fire safety. The ignition activation energies of wood samples using the Arrhenius equation were also calculated. Zachar et al. [23] published the results of an analysis focusing on the activation energy required for spontaneous ignition and flash point of Norway spruce and thermowood specimens. Karlsson and Quintere [24] stated that for flash ignition temperature phenomenon to occur, the temperature in the fire compartment should reach 500 to 600 °C, or the radiant flux on the floor should be 15 to 20 kW·m^−2^. These temperatures are significantly higher than the spontaneous ignition temperatures of most lignocellulosic materials.

The fire parameters of wood species are well known [25], but only a few scientific studies have focused on the exact assessment of changes of the key fire parameters (i.e., activation energy and burning rate) of wood and wood-based materials due to their aging.

This paper deals with the assessment of the age (0, 10, 40, 80 and 120 years) of oak wood and its chemical composition on the burning rate for the purposes of determining the causes of fires. We also investigated the relative weight loss and duration of flame burning as important characteristics when assessing materials from the perspective of fire.

## 2. Experimental

### 2.1. Material

Samples of oak wood (*Quercus robur* L.) were prepared from beams (taken from the interior part of building) of different ages (40, 80 and 120 years). The beams were obtained from historic buildings in Slovakia during their reconstruction. The age was marked on the beams and verified in the historical records of building construction. Samples prepared from 0- and 10-year-old wood were taken from wood harvested in the Zvolen locality in Slovakia.

Samples with the dimensions of 20 × 20 × 10 mm (standard STN ISO 871) [26] were conditioned at a temperature of 23 ± 2 °C and relative air humidity of 50 ± 5% for at least 40 h to the final sample moisture content of 12% (standard STN EN ISO 291) [27].

The average density of wood samples ranged from 0.641 to 0.702 g·cm^−3^ (Table 1).

### 2.2. Methods

#### 2.2.1. Chemical Composition of Wood

Samples were disintegrated into sawdust, and the size fractions 0.5 to 1.0 mm were used for the chemical analyses. According to ASTM D1107-21 [28], the extractives content was determined in a Soxhlet apparatus with a mixture of ethanol and toluene (2:1). The lignin content was determined according to Sluiter et al. [29], the cellulose according to the method by Seifert [30], and the holocellulose according to the method by Wise et al. [31]. Hemicelluloses were calculated as a difference between the holocellulose and the cellulose content. Measurements were performed on four replicates per sample. The results were presented as oven-dry wood percentages.

Elementary analysis was accomplished as follows: carbon (C) was determined according to STN ISO 10,694 [32] by using elemental analysis with thermally conductive analysis; nitrogen (N) according to ISO 13,878 [33] by using elemental analysis with thermally conductive analysis; sulfur (S) according to ISO 15,178 [34] by using elemental analysis with thermally conductive analysis; and phosphor (P), calcium (Ca), magnesium (Mg) and potassium (K) according to ISO 11,885 [35] by using atomic emission spectrometry with inductively coupled plasma.

#### 2.2.2. Flame Ignition Temperature and Spontaneous Ignition Temperatures

The flame ignition temperature (FIT) and spontaneous ignition temperature (SIT) were determined according to the STN ISO 871 standard [26]. Measurements were performed on twenty replicates per sample. The principle of the test is to heat the test material in a heating chamber at different temperatures. By positioning a small ignition flame impinging on the opening cover of the hot-air furnace, the released gases ignite and the FIT can be determined. SIT is determined the same way as the FIT, but without the igniting flame. The temperature profile in the furnace was measured using thermocouples (type K) with a diameter of 0.5 mm; the data logger ALMEMO^®^710 (Ahlborn Mess- und Regelungstechnik GmbH, Holzkirchen, Germany) was used for recording temperature. The lowest air temperature at which the sample was ignited within 10 min was recorded as the spontaneous ignition temperature. Subsequently, the induction time was found. Analysis of dependences between the induction time and the inverse values of thermodynamic temperature for the samples was performed using Statistica 12 software. The exponential equation was derived in the same software. The pre-exponential factor was further used to calculate the activation energy of spontaneous ignition. The calculation of the activation energy of spontaneous ignition (kJ·mol^−1^) was performed according to Equation (1), which is analogous to the Arrhenius equation.

(1) $$E=\ln\left(\frac{\tau }{A}\right)\times R\times T$$

where:

*τ*—induction time of spontaneous ignition (s);
*A*—pre-exponential (frequency) factor (-);
*E*—activation energy of spontaneous ignition (J·mol^−1^);
*R*—gas constant (8.314 J·K^−1^·mol^−1^);
*T*—ignition thermodynamic temperature (K).

#### 2.2.3. The Mass Burning Rate

The reaction to fire tests was determined according to the ISO 11925-2 [36] standard. The mass burning rate was measured with an instrument consisting of an electronic balance with an accuracy of two decimal places, a weight protection unit, a metal sample holder, a metal loading frame for placing the radiant heat source and an infrared thermal heater with an input of 1000 W. The sample was placed into the holder at a distance of 30 mm from the heat source for a specific time of 600 s and the weight change recorded every 10 s. The heat flux of the infrared thermal heater was 30 kW∙m^−2^. Measurements were performed on twenty replicates per sample.

To determine the burning rate in the specified time interval, the absolute burning rate υ (was calculated according to the relational Equation (2):

(2) $$\vartheta =\frac{\delta \left(\tau \right)-\delta \left(\tau +\Delta \tau \right)}{\Delta \tau }$$

where:

ϑ—absolute burning rate (%·s^−1^);
*δ* (*τ*)—specimen mass in the time (*τ*) (%);
*δ* (*τ* + Δ*τ*)—specimen mass in the time (*τ* + Δ*τ*) (%);
Δ*τ*—time interval in which the mass values are recorded (s).

## 3. Results and Discussion

### 3.1. Wood Chemical Composition

The content of the main chemical compounds, except the lignin amount, differs when comparing older and recent wood (Table 2). The proportion of the lignin does not change considerably despite the different age of the wood samples. Several authors determined the lignin content between 14.78% and 28.8% depending on the type of oak wood, zone of wood, etc. [37,38,39,40]. Kolář and Rybíček [38] analyzed subfossil oak wood (*Quercus robur* L.). Based on their results, subfossil wood contains a greater amount of fibers. In the view of chemical composition, the cellulose content does not change despite the different age of the trunk, but the lignin content is higher in subfossil wood. The cellulose and extractives comparison of our samples does display higher amounts of these components in older wood. Kačík et al. [41] observed that the cellulose in old fir wood beam samples (from 108- to 390-years-old) increased by 13%; both the lignin and holocellulose dropped by 4% compared to the recent fir wood. In contrast, the content of hemicelluloses in our samples decreased due to the aging process. Hemicelluloses are the most susceptible to degradation, even at relatively low temperatures [42,43,44]. Several authors studied wood with different natural aging time and the results confirm that the proportion of saccharides gradually decreases (mainly due to the hemicellulose degradation) and the content of lignin increases successively with increasing time [41,45,46]. According to Fengel and Wegener [47], chemical analyses of old woods show a decrease in polysaccharides and an increase in the nonhydrolyzable residues. Jebrane et al. [48] explain the degradation of polysaccharides with the presence of acetyl groups that are thermally labile and lead to the formation of acetic acid, thereby causing acid-catalyzed degradation of the polysaccharides. A more reliable indicator seems to be the cellulose to hemicelluloses ratio (C/H) [41]. In the samples of oak wood, the C/H ratio increased with age (Table 2) due to a lower stability of hemicelluloses towards cellulose.

Historic wood samples have different chemical compositions, primarily depending on the deposition conditions. The conditions determine the mechanisms and rates of wood degradation [49,50]. According to Krutul and Kocoń [51], time plays only a secondary role in the destroying process. Factors influencing wood aging include UV radiation, which has sufficient energy for photochemical degradation of structural polymer components of wood (lignin, cellulose and hemicelluloses) [52].

The results in Table 3 show the differences in elementary composition of oak wood. The 120-year-old oak wood contains the lowest amount of N, S and P, and the largest amount of C elements due to the initial carbonization process [53]. We determined a calcium content of 0.7 g·kg^−2^ in the recent wood samples, which is in accordance with the results of Krutul [54]. The amount of this element decreased with the age of wood.

Cárdenas-Gutiérrez [39] performed ash microanalysis of various oak woods with an X-ray spectrometer (*Quercus candicans*—Qc; *Quercus laurina*—Ql; *Quercus rugosa*—Qr). Based on their results, sample Qc contains 19.91% of Mg, 8.27% of P, 1.28% of S, 38.33% of K and 24.83% of Ca; sample Ql contains 5.41% of Mg, 1.22% of P, 0.22% of S, 8.93% of K and 77.11% of Ca, and sample Qr contains 27.71% of Mg, 10.34% of P, 0.97% of S, 27.07% of K and 33.28% of Ca. Kovář and Rybíček [38] conducted the analysis of the inorganic elements of subfossil wood and determined the highest content of Ca. Based on the results they assume that the samples have gone through a process of calcification.

### 3.2. Fire-Technical Properties

The spontaneous ignition temperatures together with the temperature recalculated values (inverse value of the temperature in °C to the thermodynamic temperature in K, necessary for the calculation of the activation energy) are shown in Table 4.

Based on the results given in Table 4, the sample of 10-year-old oak wood has the highest fire resistance in the view of FIT, because we marked its average temperature of 434.14 °C in a time of 257.4 s. From the point of view of fire resistance, it is important to record the highest temperature and the longest ignition time.

The results of the calculated activation energy using the Arrhenius equation are introduced. Table 5 shows the values of pre-exponential factor (A) representing the regression coefficient in the correlation equation calculated between the spontaneous ignition temperature and the induction time values of the individual samples. These data were used for the calculation of the activation energy of individual samples, including the average values of the spontaneous ignition temperature and the induction time.

The activation energy of oak samples (Table 5) with different age ranged from 42.4 kJ·mol^−1^ (120-year-old) to 50.7 kJ·mol^−1^ (40-year-old). The value of the activation energy of a 40-year-old sample was obtained in an average time of 248.2 s from the beginning of the thermal loading. Based on these results, we can state that this sample has the higher resistance against this kind of thermal loading. The data indicate that the activation energy of spontaneous ignition may become a suitable tool for a more accurate comparison of the thermal resistance of materials. The difference in spontaneous ignition temperature between these materials is minimal (according to ISO 871:2006, the ignition temperature is measured with an accuracy of 10 °C), but the burning time achieved was different. The difference in the activation energy of spontaneous ignition is very significant.

The lowest thermal resistance of oak wood was observed in the oldest wood (120-year-old), which could cause its degradation and decomposition of the main chemical components. The higher content of extractives in this sample indicates the decomposition of lignin as the most stable macromolecule [55,56]. This sample also has the lowest proportion of saccharides, mainly due to the degradation of hemicelluloses (C/H ratio = 1.09). Several authors describe the decrease of polysaccharides during natural aging of wood [45,57]. Therefore, older wood poses a greater risk of ignition compared to the younger oak wood.

In general, the values of the induction time (τ) decrease with increasing values of the temperatures (t) [23].

The scientific work of Tureková and Balog [58] showed that the activation energy of spontaneous ignition, even if the sample weight is up to one gram, significantly depends on the weight of the sample. The solution for obtaining the spontaneous ignition activation energy from these external factors is the measure the activation energy of spontaneous ignition for a particular sample and the specific conditions (e.g., modeling the dynamics of development of a particular fire, investigation of a specific fire, etc.).

**Figure 1:**
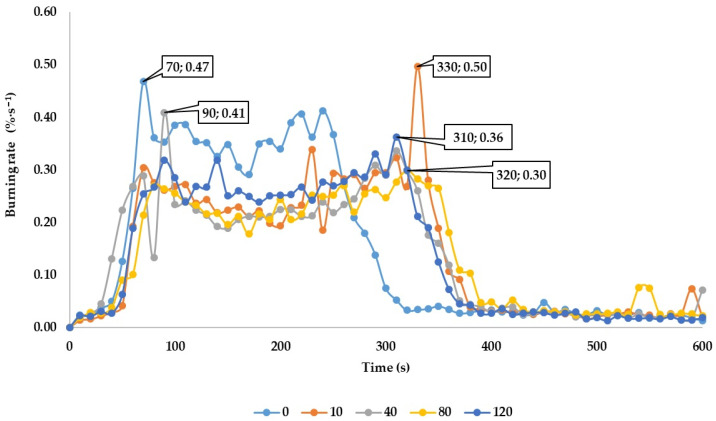
The absolute burning rate of the oak wood.

The results of the mass burning rate are shown in Figure 1, where the maximum values of burning rate and the time needed to reach the maximum values of burning rate are given.

The maximum values of burning rate and the time needed to reach the maximum values of burning rate are presented in Figure 1.

Based on the changes in the mass burning rate curves shown in Figure 1, it can be stated that the maximum burning rates can be observed over the entire time interval during which the samples were subjected to the thermal loading.

The maximum burning rate of 0.4965%·s^−1^ was reached in 330 s for 10-year-old samples. This was followed by 0-year-old samples, where the maximum burning rate was 0.4678%·s^−1^ that was reached in 70 s; 40-year-old samples with the burning rate of 0.4093%·s^−1^ that was reached in 90 s and 120-year-old samples with the burning rate of 0.3625%·s^−1^ that was reached in 320 s. The lowest burning rate of 0.2992%·s^−1^ was reached by 80-year-old samples in 320 s from the beginning of the thermal load. Based on the results of burning rates, no clear conclusion can be drawn about the influence of the age of the samples on the burning rate. The results show that the initiation and the subsequent thermal degradation of oak wood occur in time interval from 30 to 90 s from the beginning of the thermal loading. The impact of the age of the samples on initiation and subsequent development of the fire cannot be confirmed.

The reason for a lower rate values was the difference in the initial phase of sample burning. Kačíková a Makovická [59] compared coniferous species. The lowest average burning rate, 0.090%·s^−1^, was reached for spruce wood. The maximum burning rate for spruce wood reached 0.187%·s^−1^ in 180 s from the beginning of the thermal load.

## 4. Conclusions

The content of extractives and cellulose is higher and hemicelluloses lower with increasing age of oak wood. The proportion of lignin does not change.

The 120-year-old samples contain the lowest relative content of hemicelluloses and the highest relative content of both lignin and extractives due to the degradation processes.

From the perspective of elementary analysis, the 120-year-old oak wood sample contains the highest amount of carbon due to the initial carbonization process and the lowest amount of nitrogen, sulfur and phosphor.

Samples of 10-year-old oak wood have the greatest fire resistance in the view of FIT.

In terms of fire-technical properties, especially the highest activation energy reached, 50.684 kJ·mol^−1^, we can state that the 40-year-old oak wood samples have the highest thermal resistance.

The maximal burning rate values are range from 0.2992%·s^−1^ (80-year-old oak wood samples) to 0.4965%·s^−1^ (10-year-old oak wood samples).

Comparing the activation energy (49.535 kJ·mol^−1^) and the mass burning rate (0.2992%·s^−1^), the 80-year-old samples have the greatest fire resistance. Samples of 120-year-old oak wood have the lowest values of these parameters; these samples also contain the lowest amount of N, S, P and the largest amount of C elements due to the initial carbonization process.

## Figures and Tables

**Table 1:** The average density of oak wood samples.

Approximate Age of Oak Sample (years)	Density (g·cm^−3^)
recent	0.681 ± 0.03
10	0.641 ± 0.11
40	0.660 ± 0.28
80	0.688 ± 0.12
120	0.702 ± 0.21

**Table 2:** Relative content of the main chemical components of oak wood.

Age of Oak Sample (years)	Extractives (%)	Lignin (%)	Cellulose (%)	Holocellulose (%)	Hemicelluloses (%)	C/H Ratio
0	3.93 ± 0.04	23.04 ± 0.14	33.48 ± 0.06	73.03 ± 0.11	39.55 ± 0.17	0.84
10	3.97 ± 0.06	22.86 ± 0.04	33.79 ± 0.57	73.16 ± 0.02	39.37 ± 0.55	0.86
40	5.78 ± 0.04	22.14 ± 0.02	33.41 ± 0.11	72.08 ± 0.06	38.67 ± 0.23	0.86
80	6.62 ± 0.03	22.31 ± 0.02	36.22 ± 0.01	71.08 ± 0.04	34.85 ± 0.03	1.04
120	7.34 ± 0.11	22.91 ± 0.04	36.40 ± 0.08	69.75 ± 0.15	33.35 ± 0.23	1.09

**Table 3:** Elementary analysis of oak wood.

Age of Oak Sample (years)	Carbon (g·kg^−2^)	Nitrogen (g·kg^−2^)	Sulfur (mg·kg^−2^)	Phosphor (g·kg^−2^)	Calcium (g·kg^−2^)	Magnesium (g·kg^−2^)	Potassium (g·kg^−2^)
0	489	1.32	299	0.151	0.702	0.044	0.621
10	491	1.07	252	0.155	0.495	0.134	0.921
40	486	1.35	291	0.145	0.296	0.029	0.834
80	493	1.29	122	0.14	0.244	0.008	0.435
120	501	0.98	94	0.118	0.293	0.022	0.544

**Table 4:** Flame ignition temperature and spontaneous ignition temperature of oak wood samples.

Age of Oak Sample (years)	Thermal Loading	Average Time τ (s)	Average Temperature t (°C)	Average Temperature T (K)	Inverse Value 1/T (K^−1^)
0	FIT	219.6 ± 21.38	436.02 ± 21.05	709.17	0.0014111
	SIT	336.4 ± 18.49	374.96 ± 23.09	648.11	0.0015448
10	FIT	257.4 ± 13.33	434.14 ± 23.14	706.19	0.0014153
	SIT	336.1 ± 19.51	375.43 ± 24.30	648.58	0.0015436
40	FIT	248.2 ± 25.28	432.43 ± 17.62	705.58	0.0014182
	SIT	376.1 ± 20.29	371.24 ± 26.72	644.39	0.0015542
80	FIT	221.6 ± 17.14	429.52 ± 16.24	702.67	0.0014274
	SIT	379.2 ± 17.58	361.23 ± 22.13	633.33	0.0015777
120	FIT	229.5 ± 24.30	430.48 ± 18.12	703.63	0.0014219
	SIT	391.2 ± 19.54	363.37 ± 21.89	636.52	0.0015727

**Table 5:** The activation energy values of individual test samples.

Age of Oak Sample (years)	Exponential Equation	τ (s)	A	Activation Energy (kJ·mol^−1^)
0	y = 0.067 × e^5715.9x^	219.6	0.0672	47.710
10	y = 0.108 × e^5472x^	257.4	0.1080	45.727
40	y = 0.044 × e^6077x^	248.2	0.0439	50.684
80	y = 0.045 × e^5941x^	221.6	0.0450	49.535
120	y = 0.162 × e^5090.9x^	229.5	0.1621	42.444

## References

[1] EN 1995-1-1 + A1 (2008). Eurocode 5. Design of Wooden Structures. Depending on the Required Fire Resistance of the Building Structure, the Minimum Cross-Sectional Dimensions of Load-Bearing Wooden Elements in Wooden Structures Must Be Designed.

[2] Kačíková D., Majlingová A., Veľková V., Zachar M. (2017). Modelling of Internal Fires Using the Results of Progressive Methods of Fire Engineering.

[3] Friquin K.L. (2010). Material properties and external factors influencing the charring rate of solid wood and glue-laminated timber. Fire Mater..

[4] Cachim P.B., Franssen J.-M. (2010). Assessment of Eurocode 5 Charring Rate Calculation Methods. Fire Technol..

[5] White R., Dietenberger M. (2001). Wood Products: Thermal Degradation and Fire. Encyclopedia of Materials: Science and Technology.

[6] Bartlett A.I., Hadden R.M., Bisby L.A. (2019). A Review of Factors Affecting the Burning Behaviour of Wood for Application to Tall Timber Construction. Fire Technol..

[7] Lau P.W.C., White R., Van Zeeland I. (1999). Modelling the charring behaviour of structural lumber. Fire Mater..

[8] Očkajová A., Kučerka M., Kminiak R., Krišťák Ľ., Igaz R., Réh R. (2020). Occupational exposure to dust produced when milling thermally modified wood. Int. J. Environ. Res. Public Health.

[9] Aristri M., Lubis M., Yadav S., Antov P., Papadopoulos A., Pizzi A., Fatriasari W., Ismayati M., Iswanto A. (2021). Recent Developments in Lignin- and Tannin-Based Non-Isocyanate Polyurethane Resins for Wood Adhesives—A Review. Appl. Sci..

[10] Kačíková D., Kubovský I., Ulbriková N., Kačík F. (2020). the impact of thermal treatment on structural changes of teak and iroko wood lignins. Appl. Sci..

[11] Frangi A., Fontana M. (2003). Charring rates and temperature profiles of wood sections. Fire Mater..

[12] Njankouo J.M., Dotreppe J.-C., Franssen J.-M. (2004). Experimental study of the charring rate of tropical hardwoods. Fire Mater..

[13] Schmid J., Just A., Klippel M., Fragiacomo M. (2015). The Reduced Cross-Section Method for Evaluation of the Fire Resistance of Timber Members: Discussion and Determination of the Zero-Strength Layer. Fire Technol..

[14] Sonderegger W., Kránitz K., Bues C.-T., Niemz P. (2015). Aging effects on physical and mechanical properties of spruce, fir and oak wood. J. Cult. Herit..

[15] Kránitz K., Sonderegger W., Bues C.-T., Niemz P. (2016). Effects of aging on wood: A literature review. Wood Sci. Technol..

[16] Topaloglu E., Ustaomer D., Ozturk M., Pesman E. (2021). Changes in wood properties of chestnut wood structural elements with natural aging. Maderas Cienc. Tecnol..

[17] Reinprecht L. (2016). Wood Deterioration, Protection and Maintenance.

[18] Majlingová A., Zachar M., Lieskovský M., Mitterová I. (2018). The analysis of mass loss and activation energy of selected fast-growing tree species and energy crops using the Arrhenius equation. Acta Fac. Xylologiae Zvolen.

[19] Martinka J., Mózer V., Hroncová E., Ladomerský J. (2015). Influence of spruce wood form on ignition activation energy. Wood Res..

[20] Rantuch P., Wachter I., Hrušovský I., Balog K. (2016). Ignition Activation Energy of Materials based on Polyamide 6. Trans. VSB Tech. Univ. Ostrav. Saf. Eng. Ser..

[21] Martinka J., Hroncová E., Kačíková D., Rantuch P., Balog K., Ladomerský J. (2017). Ignition parameters of poplar wood. Acta Fac. Xylologiae.

[22] Luptáková J., Kačík F., Eštoková A., Kačíková D., Šmíra P., Nasswettrová A., Bubeníková T. (2018). Comparison of activation energy of thermal degradation of heat sterilised silver fir wood to larval frass regarding fire safety. Acta Fac. Xylologiae Zvolen.

[23] Zachar M., Majlingová A., Šišulák S., Baksa J. (2017). Comparison of the activation energy required for spontaneous ignition and flash point of the Norway spruce wood and thermowood specimens. Acta Fac. Xylologiae Zvolen.

[24] Karlsson B., Quintiere J. (1999). Enclosure Fire Dynamics.

[25] Shi L., Chew M.Y.L. (2012). Experimental study of woods under external heat flux by autoignition. J. Therm. Anal. Calorim..

[26] STN ISO 871 (2010). Plastics. Determination of Ignition Temperature Using a Hot-Air Oven.

[27] STN EN ISO 291 (2008). Plastics. Standard Atmospheres for Conditioning and Testing.

[28] ASTM D1107-21 (2021). Standard Test Method for Ethanol-Toluene Solubility of Wood.

[29] Sluiter A., Hames B., Ruiz R., Scarlata C., Sluiter J., Templeton D., Crocker D. (2012). Determination of Structural Carbohydrates and Lignin in Biomass (NREL/TP-510-42618).

[30] Seifert V.K. (1956). About a new method for rapid determination of pure cellulose. Das Pap..

[31] Wise L.E., Murphy M., D’addieco A.A. (1946). Chlorite holocellulose, its fractionation and bearing on summative wood analysis and on studies on the hemicelluloses. Pap. Trade J..

[32] ISO 10694 (1995). Soil Quality. Determination of Organic and Total Carbon after Dry Combustion (Elementary Analysis).

[33] ISO 13878 (1998). Soil Quality. Determination of Total Nitrogen Content by Dry Combustion (Elemental Analysis).

[34] ISO 15178 (2000). Soil Quality. Determination of Total Sulfur by Dry Combustion.

[35] ISO 11885 (2007). Water Quality. Determination of Selected Elements by Inductively Coupled Plasma Optical Emission Spectrometry (ICP-OES).

[36] ISO 11925-2 (2020). Reaction to Fire Tests—Ignitability of Products Subjected to Direct Impingement of Flame—Part 2: Single-Flame Source Test.

[37] Santos R.B., Capanema E.A., Balakshin M.Y., Chang H., Jameel H. (2012). Lignin structural variation in hardwood species. J. Agric. Food Chem..

[38] Kolář T., Rybníček M. (2014). The changes in chemical composition and properties of subfossil oak deposited in holocene sediments. Wood Res..

[39] Cárdenas-Gutiérrez M.A., Pedraza-Bucio F.E., López-Albarrán P., Rutiaga-Quiñones J.G. (2018). Chemical components of the branches of six hardwood species. Wood Res..

[40] Hrčka R., Kučerová V., Hýrošová T. (2018). Correlations between oak wood properties. BioResources.

[41] Kačík F., Šmíra P., Kačíková D., Reinprecht L., Nasswettrova A. (2014). Chemical changes in fir wood from old buildings due to ageing. Cellul. Chem. Technol..

[42] Kučerová V., Lagaňa R., Výbohová E., Hýrošová T. (2016). The effect of chemical changes during heat treatment on the color and mechanical properties of fir wood. BioResources.

[43] Kubovský I., Kačíková D., Kačík F. (2020). Structural Changes of Oak Wood Main Components Caused by Thermal Modification. Polymers.

[44] Čabalová I., Zachar M., Kačík F., Tribulová T. (2019). Impact of thermal loading on selected chemsical and morphological properties of spruce ThermoWood. BioResources.

[45] Popescu C.-M., Hill C.A.S. (2013). The water vapour adsorption–desorption behaviour of naturally aged *Tilia cordata* Mill. wood. Polym. Degrad. Stab..

[46] Zhao C., Zhang X., Liu L., Yu Y., Zheng W., Song P. (2019). Probing Chemical Changes in Holocellulose and Lignin of Timbers in Ancient Buildings. Polymers.

[47] Fengel D., Wegener G. (1989). Wood—Chemistry, Ultrastructure, Reactions.

[48] Jebrane M., Pockrandt M., Cuccui I., Allegretti O., Uetimane E., Terziev N. (2018). Comparative Study of two softwood species industrially modified by Thermowood (R) and thermo-vacuum process. BioResources.

[49] Passialis C.N. (1977). Physico-chemical characteristics of waterlogged archaeological wood. Holzforschung.

[50] Florian M.L.E., Rowell R.M., Barbour R.J. (1990). Scope and history of archaeological wood. Archaeological Wood: Properties, Chemistry, and Preservation.

[51] Krutul D., Kocoń J. (1982). Inorganic constituents and scanning electron microscopic study of fossil oak wood (*Quercus* sp.). Holzforsch. Holzverwendung.

[52] Teaca C.A., Roşu D., Mustaţă F., Rusu T., Roşu L., Roşca I., Varganici C.D. (2019). Natural bio-based products for wood coating and protection against degradation: A Review. BioResources.

[53] Carrión J.S., Marin D. (2003). Plant Evolution (Evolución Vegetal).

[54] Krutul D., Radomski A., Zawadzki J., Zielenkiewicz T., Antczak A. (2010). Comparison of the chemical composition of the fossil and recent oak wood. Wood Res..

[55] Wikberg H., Maunu S.L. (2004). Characterisation of thermally modified hard- and softwoods by 13C CPMAS NMR. Carbohydr. Polym..

[56] Čabalová I., Kačík F., Lagaňa R., Výbohová E., Bubeníková T., Čaňová I., Ďurkovič J. (2018). Effect of thermal treatment on the chemical, physical, and mechanical properties of pedunculate oak (*Quercus robur* L.) wood. BioResources.

[57] Čabalová I., Bélik M., Kučerová V., Jurczyková T. (2021). Chemical and Morphological Composition of Norway Spruce Wood (*Picea abies*, L.) in the Dependence of its Storage. Polymers.

[58] Tureková I., Balog K. (2001). Flame ignition parameters of polyethylene and activation energy of initiation of combustion process. Res. Pap..

[59] Kačíková D., Makovická-Osvaldová L. (2009). Wood burning rate of various tree parts from selected softwoods. Acta Fac. Xylologiae Zvolen.

